# Optimized Depilation Method and Comparative Analysis of Hair Growth Cycle in Mouse Strains

**DOI:** 10.3390/ani14142131

**Published:** 2024-07-22

**Authors:** Joana Magalhaes, Sofia Lamas, Carlos Portinha, Elsa Logarinho

**Affiliations:** 1 Insparya Science and Clinical Institute, 4150-516 Porto, Portugal; carlos.portinha@insparya.com (C.P.); elsa.logarinho@i3s.up.pt (E.L.); 2 Aging and Aneuploidy Group, i3S-Instituto de Investigação e Inovação em Saúde, Universidade do Porto, 4200-135 Porto, Portugal; 3 Animal Facility, i3S-Instituto de Investigação e Inovação em Saúde, Universidade do Porto, 4200-135 Porto, Portugal

**Keywords:** hair cycle, depilation method, androgenic alopecia, animal model, animal welfare

## Abstract

**Simple Summary:**

Depilation is a well-established method for synchronizing the hair growth in mice which is valuable to test hair growth interventions. However, our experience with the previously published depilation protocols showed that upon depilation mice developed wounds. Here, we propose a new depilation method that protects the mouse skin, minimizing skin injuries and improving not only experimental conditions but also animal welfare. We test this new protocol not only in *C57BL/6J* mice, the most commonly used strain in hair growth pre-clinical studies, but also in other two strains (*Sv129* and *B6129F1)*, which lack a well-characterized hair growth cycle. Our results show that hair cycle kinetics are similar in all the analyzed strains, but *Sv129* and *B6129F1* skins are morphologically different from *C57BL/6J* skin, presenting increased number and size of hair follicles in anagen, consistent to the higher hair density observed macroscopically. This study reveals a new method to improve animal hair research and characterizes the hair growth in two different strains, enhancing their suitability in future studies.

**Abstract:**

In mice, hair growth follows a mosaic or wavy patterning. Therefore, synchronization of the hair growth cycle is required to adequately evaluate any trichogenic interventions pre-clinically. Depilation is the established method for synchronizing the growth phase of mouse hair follicles. When attempting to reproduce procedures reported in the literature, *C57BL/6J* mice developed severe wounds. This led us not only to optimize the procedure, but also to test the procedure in other strains, namely *Sv129* and the F1 generation from *C57BL/6J* crossed with *Sv129* (*B6129F1* mixed background), for which the hair growth cycle has not been ascertained yet. Here, we describe an optimized depilation procedure, using cold wax and an extra step to protect the animal skin that minimizes injury, improving experimental conditions and animal welfare in all strains. Moreover, our results show that, although hair cycle kinetics are similar in all the analyzed strains, *Sv129* and *B6129F1* skins are morphologically different from *C57BL/6J* skin, presenting an increased number and size of hair follicles in anagen, consistent to the higher hair density observed macroscopically. Altogether, the results disclose an optimized mouse depilation method that excludes the detrimental and confounding effects of skin injury in hair growth studies and reveals the hair cycle features of other mouse strains, supporting their use in hair growth pre-clinical studies.

## 1. Introduction

Mouse models have been extensively used in hair research, not only for basic research on molecular mechanisms of hair follicle morphogenesis and growth cycle, but also for testing new pharmacological and bioengineering treatments for hair loss.

Notwithstanding the many differences between human and mouse skin, several morphogenic and regenerative signaling pathways are still conserved, which allows for the use of mouse models in hair research [1]. The *C57BL/6J* mouse strain has been largely used for dermatologic studies due to the black pigmentation of the hair in contrast to a light skin, which facilitates trichoscopy analysis. For this reason, the hair cycle timeline has been well characterized in this strain [2]. The hair growth cycle consists of three phases: a resting telogen phase, an anagen phase of active hair growth, and finally, a catagen phase when hair growth stops, leading to the telogen phase of another cycle [2]. As *C57BL/6J* mice epidermis lacks melanin-producing melanocytes, their skin pigmentation is exclusively dependent on their follicular melanocytes that are only active during anagen. Thus, the hair cycle stages can be easily identified in this mouse strain based on the alternating gray and pink skin colors [2,3].

In a population of hair follicles, each hair follicle proceeds through its own cycle stages independently of its neighboring hair follicles, explaining the mosaic or wavy patterning of hair growth across the whole body of the mouse [4]. For this reason, synchronization of the hair follicle growth cycles is used for studies in mouse models. Depilation is a well-established method for synchronizing the growth phase of hair follicles as it triggers the transition from the resting telogen phase to the active anagen phase. Both depilatory cream and wax have been used to this end. However, depilatory creams tend to leave mild skin irritations [5] and do not completely remove the hair follicle. Depilation by warm rosin or wax [6,7] or by cold wax strips [8,9] allows for the complete removal of the hair follicle, which is essential for hair regeneration studies. When we attempted to reproduce the literature procedures, using warm wax or cold wax strips, the mice developed wounds, which are known to affect hair growth [10,11] and impair animal welfare during experiments. This issue led us to optimize the depilation procedure as described in this article.

Although the *C57BL/6J* mice are the mostly used for hair research, this strain is acknowledged to be prone to skin conditions such as ulcerative dermatitis [12]. The development of wounds after depilation led us to, in addition to optimizing the procedure, test the procedure in other mice strains as detailed in this article. Notably, the literature lacks a well-defined characterization of hair growth in other strains. Therefore, we complemented our study by resorting to another mouse strain with dark hair, *Sv129*, as well as to the F1 generation of *C57BL/6J* crossed with *Sv129* (*B6129F1*), often present in genetically modified mouse models used in research.

Pre-clinical studies in regenerative therapies for hair loss rely on mouse models and depilation methodologies. However, we found that a detailed description of the procedures is largely missing, as well as, in our hands, the previous procedures often led to skin lesions. Moreover, limited information is found for *Sv129* and *B6129F1* strains. To mitigate this, here we present a detailed and improved protocol for mouse depilation with a negligible impact on the mouse skin. This protocol was tested on different mouse strains, and a comparative analysis for their hair growth cycle was performed.

## 2. Methods

### 2.1. Mice

*C57BL/6J* mice (male and female) were purchased from the Charles River Laboratories, while *B6129F1* and *Sv129* (*129S2/SvPasCrl*) mice were breed in house at the i3S animal facility. Six-week-old mice were housed and allowed to adapt to the environment for one week before experiments started. Animals were maintained under a 12 h light/dark cycle and fed with regular sterilized rodent’s chow (Envigo 2014S) and tap water ad libitum, at the i3S animal facility. Mice were maintained inside Eurostandard type II cages, in groups of 3 to 4 animals. Environmental enrichment consisted of paper as nesting material and tube role. All animals had specific pathogen free sanitary status (SPF), according to the FELASA recommendations, with occasional positives for *Staphylococcus aureus*. Studies were conducted in compliance with institutional ethical guidelines (i3S Animal Welfare and Ethics Review Body, ORBEA) and with the National and European Union rules (2010/63/EU), under DGAV licensing. Table 1 details the total number of animals used. When possible, these mice were shared and used in other studies.

### 2.2. Optimized Depilation Method

Seven-week-old *C57BL/6J*, *B6129F1* or *Sv129* male and female mice were used. Hair growth cycle synchronization was induced by depilation. Mice were anesthetized using 3% isoflurane in 0.3–0.4 L/min O_2_ and kept in a heating pad during the depilation procedure. The backs of the mice were shaved using a hair shaver (ISIS, B. Braun, Melsungen, Germany) and depilated using cold wax bands (*Veet Minima*). Shaving was performed in a wider region (3 × 2 cm) followed by depilation in a smaller rectangular area (2 × 1.5 cm) to avoid wax sticking to grown non-shaved hair. The cold wax stripe was applied once with the skin well stretched and removed at once against the direction of hair growth. After the depilation, to protect the skin of the depilated area, the excess of wax was removed using mineral oil (Sigma, St. Louis, MA, USA, M8410), and a skin protector spray, Douxo Calm Sérum (Ceva, Libourne, France), was applied, followed by Cavilon (3M, St. Paul, MN, USA), a barrier film spray.

In parallel, other methods of depilation were performed. For this, mice were anesthetized, shaved with a hair shaver, as described previously, then depilated using warm wax; warm wax followed by wiping the area with mineral oil to remove excess wax; cold wax bands (*Veet Minima*) alone; cold wax bands (*Veet Minima*) plus cleaning with an oil wipe (*Veet Minima*); cold wax bands (*Veet Minima*) followed by cleaning the area with mineral oil. The wax was applied once with the skin well stretched and removed at once against the direction of hair growth. Mice were then allowed to recover in the heating pad and returned to their cages.

### 2.3. Quantification of Wound Development

**Figure 1:**
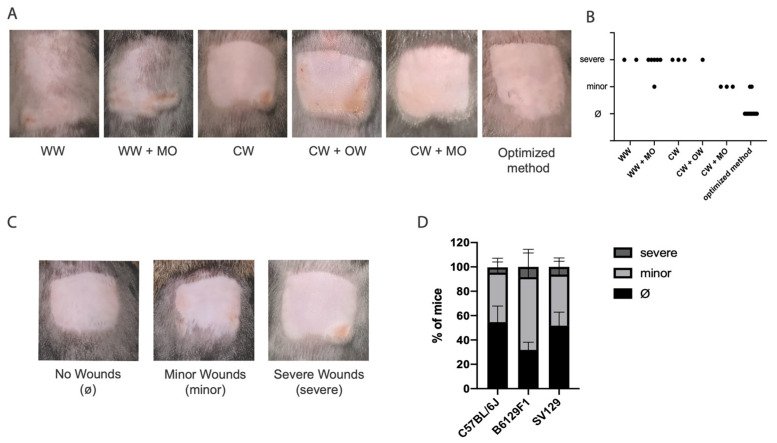
**A new optimized protocol minimizing skin injury during mouse depilation.** (**A**) Photographs of mouse skin after depilation with warm wax (WW), warm wax followed by clearance with mineral oil (WW + MO), (C) cold wax (CW), cold wax followed by clearance with a commercial oil wipe (CW + OW), cold wax followed by clearance with mineral oil (CW + MO), and our optimized method. (**B**) Distribution of wound severity in optimized (n = 10) vs. each of the other methods (n = 15). Dots correspond to individual mice. Wound severity was classified as no wounds (Ø, when no wounds were present), minor wounds (minor, when only erythema and/or superficial/erosive lesions where present) or deep wounds (severe, skin with several erosion sites and/or ulcerative areas). (**C**) Representative images for Ø, minor and severe wounding, and accordingly to classification used for quantifications in (**B**,**D**). (**D**) Quantification of wound severity using the new optimized protocol in *C57BL/6J*, *B6129F1* and *Sv129.* Values are mean ± s.d. of 3–4 independent experiments for each strain, *C57BL/6J* (total of n = 19 mice), *B6129F1* (total of n = 17 mice) and *Sv129* (total of n = 17 mice). No significant differences were observed in depilation outcomes in the different strains using two-way ANOVA with Tukey’s multiple-comparison correction statistical test (**C**).

As wound development took 1 to 2 days, wound quantification was analyzed at day 2 post-depilation. For wound quantification and for comparative purposes, seven-week-old *C57BL/6J*, *B6129F1* and *Sv129* mice were photographed with a standard camera two days after depilation, and visually characterized. Wound development and progression were assessed by the animal house veterinary. Wound severity (Figure 1C) was classified as: no wounds, minor wounds (when only erythema and/or superficial/erosive lesions where present, healing time < 24 h) or severe wounds (skin with several erosion sites and/or ulcerative areas, healing time > 24 h). When needed, treatment with iodine was applied to guarantee animal welfare.

### 2.4. Hair Growth Analysis

For hair growth analysis in different mouse strains, 7-week-old *C57BL/6J*, *B6129F1* and *Sv129* mice were depilated using our optimal protocol (described above). Hair growth was followed for 20 days by photographing the mice with a standard camera.

### 2.5. Hair Cycle Analysis

For hair cycle analysis the dorsal skin from the depilated region of the mice was collected at different timepoints. Skin was fixed in 10% buffered formalin, imbedded in paraffin and sectioned along the transversal and longitudinal plan of the hair follicles (3 µm sections). A standard protocol was used for hematoxylin and eosin staining [13]. Images were acquired under an Olympus light microscope, with a 5× objective, a DP 25 Camera and the software Cell B v3.4 (Olympus Corporation, Tokyo, Japan). n ≥ 4 field views were quantified per animal. Total skin thickness was quantified by measuring the length from epidermis to the muscular layer, which included epidermis, dermis, and subcutaneous layer. The subcutaneous layer thickness, number of hair follicles, and size of hair follicles (diameter) were also quantified. Quantifications were performed using the length measurement tool or the cell count tool available in the opensource software ImageJ/Fiji v1.52p.

### 2.6. Statistical Analysis

Statistical analysis was performed using Prism^®^ 8 software (GraphPad, Boston, MA, USA). Sample sizes are indicated in the legend of each figure. Normal distribution of the data was tested using the Shapiro–Wilk test, and the appropriate statistical test was used according to the data distribution and indicated in the figure legend. *p* < 0.05 is considered as significant. The reported results follow the ARRIVE guidelines.

## 3. Results

### 3.1. An Optimized Depilation Protocol Minimizing Skin Injury

Depilation is commonly used in hair research for synchronization of the growth phase of mice hair follicles. Both warm wax and cold wax have been reported [2,6,8,9]; however, the procedure steps and outcome have not been described in detail. Using 7-week-old *C57BL/6J* mice, the strain mostly used in hair research, we attempted to reproduce the depilation procedures using warm wax and cold wax strips (Figure 1A). We started by shaving the back of each mouse using an electric shaver. We then proceeded with depilation using either warm wax or cold wax strips. In both cases the mice developed superficial wounds (erosions) and erythema the day after depilation. Some animals also developed deeper wounds, with ulcerative areas in the depilated zone (Figure 1A: WW, WW + MO, CW, CW + OW). It is worth mentioning that the warm wax procedure was not as efficient in removing the hair as the cold wax strips, leaving hair in the depilated area (Figure 1A: WW and WW + O vs. CW). Since with the tested methods all mice were invariably developing minor and severe wounds (Figure 1A), we went on optimizing the depilation procedure. First, we used mineral oil to remove the excess of wax stuck to the skin and possibly contributing to wounds. In the case of warm wax this did not improve the outcome; however, in the case of the cold wax the use of mineral oil led to a substantial decrease in the development of wounds, but the skin still looked dry. Therefore, after depilation with cold wax and cleaning with mineral oil, we applied a protective and regenerative spray, Douxo Calm Sérum, followed by Cavilon, a barrier film spray. These optimization steps substantially improved the appearance of the skin and decreased the incidence of wounds in mice (Figure 1B). Overall, whereas the other procedures led to 100% wound frequency (most of them severe), the new optimized protocol decreased wound frequency to half (Figure 1B). Notably, only erythema and smaller erosions were observed, typically on the edge of the depilation area, with short healing time (24 h) (minor wounds, Figure 1C), For comparative purposes we applied our new optimized protocol to other mice strains, namely *B6129F1* and *Sv129*, which presented similar results (Figure 1D).

### 3.2. Hair Growth Cycle in Distinct Mouse Strains

**Figure 2:**
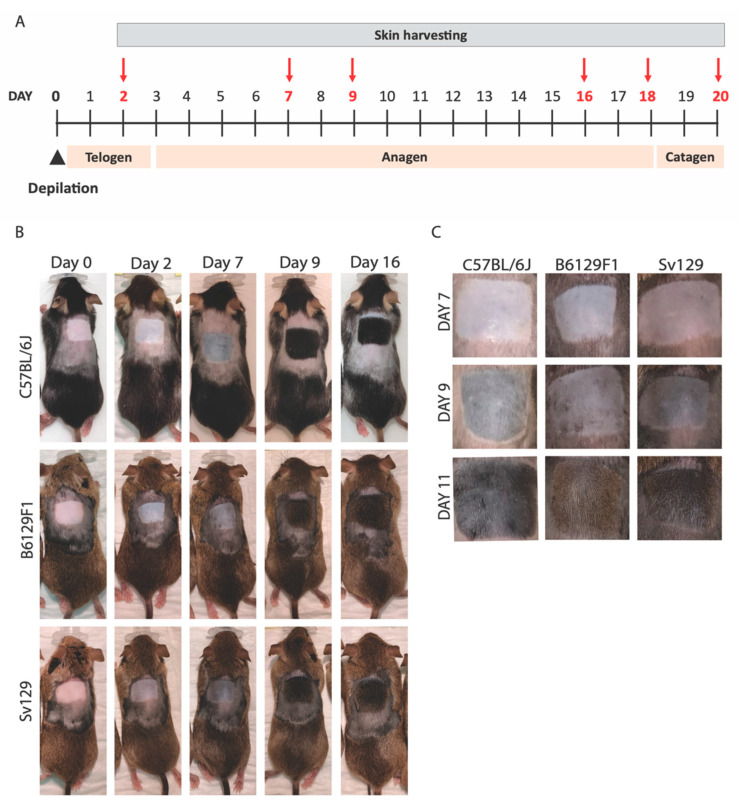
**Comparative analysis of hair growth in different mouse strains**. (**A**) Scheme of hair growth cycle time-scale in 7-week-old *C57BL/6J* depilated mice; timepoints used for tissue collection are highlighted in red. (**B**) Photographs of *C57BL/6J*, *B6129F1* and *Sv129* mice at distinct days post-depilation as indicated. (**C**) Magnified photographs of the depilation site of *C57BL/6J*, *B6129F1*, and *Sv129* mice. N = 3–5 mice were used per timepoint per strain for images and skin collection.

In 7-week-old *C57BL/6J*, hair follicles are synchronized to telogen after depilation, entering anagen simultaneously across the whole depilated area, following a well described hair cycle [2] (Figure 2A). Our analysis of the depilated area of 7-week-old *C57BL/6J* mice revealed the expected timeline of skin color change and hair growth, that is, two days after depilation mice skin was still pink, changing to gray around day 7, followed by hair growth from that point onwards (Figure 2B,C). Comparing this time-scale to the *Sv129* and *B6129F1* mice, again at two days after depilation the mice presented pink skin, that became darker around day 7, with hair growth starting from that point onwards (Figure 2B,C). However, at days 9–11 post-depilation, *Sv129* mice presented darker skin, suggesting a higher hair density compared to *C57BL/6J* (Figure 2B,C).

**Figure 3:**
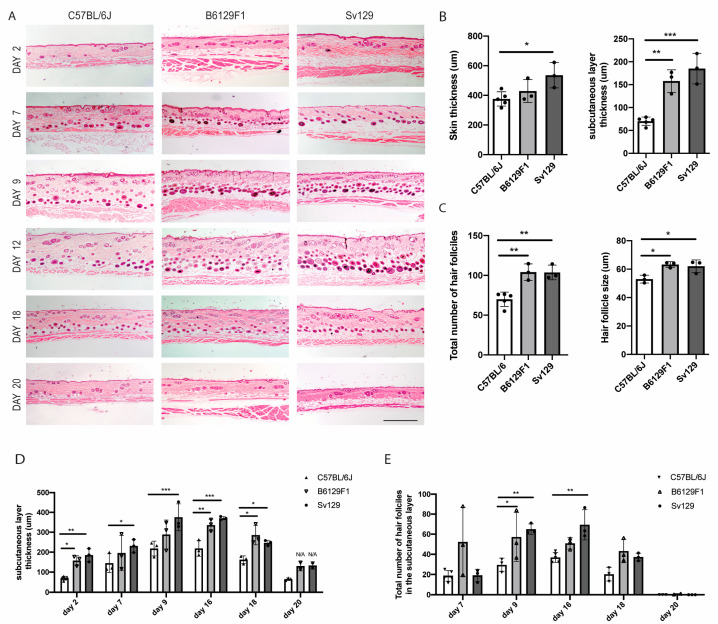
**Histomorphological differences between mouse strains.** (**A**) Hematoxilin-eosin staining of dorsal skin transversal paraffin sections from *C57BL/6J*, *B6129F1*, and *Sv129* mice at different hair cycle timepoints. Scale bar: 500 μm. (**B**) Quantification of whole skin thickness and subcutaneous layer thickness in telogen (day 2). (**C**) Quantification of the total number of hair follicles per field view and of hair follicle size in anagen (day 16). (**D**,**E**) Quantification of the (**D**) subcutaneous layer thickness and (**E**) total number of hair follicles in the subcutaneous layer (anagenic) at different timepoints across the hair cycle. In total, 3 to 5 mice were analyzed per timepoint per strain, and n ≥ 4 field views were quantified per animal. Dots in graphs correspond to individual animals. Values are mean ± s.d. Statistics were performed using one-way ANOVA, with Tukey´s multiple-comparison correction (**B**,**C**) or two-way ANOVA with Tukey´s multiple-comparison correction (**D**,**E**), * *p* < 0.05; ** *p* < 0.01, *** *p* < 0.001.

Next, we investigated the histomorphological differences between these mice strains (Figure 3A). We observed that, in telogen, the skin of the *Sv129* mice is significantly thicker than in the other strains, and that the subcutaneous layer is thicker in both *B6129F1* and *Sv129* in comparison to *C57BL/6J* (Figure 3B). When in anagen, both *B6129F1* and *Sv129* mice presented an increased number of total hair follicles as well as increased hair follicle size (Figure 3C).

To further analyze the differences between *C57BL/6J*, *B6129F1* and *Sv129* hair cycle, we followed the skin histomorphological changes through time. The subcutaneous layer becomes thicker as the hair cycle progresses through anagen, then shrinking in catagen. The subcutaneous layer thickness in *B6129F1* and *Sv129* is consistently higher than in *C57BL/6J*, indicating physiological differences between the mouse strains. Still, in all strains, the thickness of this layer responds, as expected, to the hair cycle stage, enlarging in anagen and shortening in catagen/telogen (Figure 3D).

Regarding the number of hair follicles in anagen, *B6129F1* and *Sv129* strains present more hair follicles in anagen compared to *C57BL/6J* (Figure 3E). At day 18 post-depilation, when catagen should be proceeding, *B6129F1* and *Sv129* mice still had more hair follicles in the subcutaneous layer, although most of them in catagen (notice that the hair follicles are all aligned in the border with the dermis). Two days later, at day 20, the hair cycle was concluded (back to telogen) in all strains (Figure 3A).

Altogether our data indicate that *B6129F1* and *Sv129* strains exhibit an enlarged subcutaneous layer with an increased hair follicle number and size in anagen, which is in line with the higher hair density observed macroscopically (Figure 2). Yet, the timeline of the hair growth cycle seems similar in all strains.

## 4. Discussion

Synchronization of the hair growth cycle in mouse models through depilation is an essential step for hair research, as mice have a natural mosaic or wavy patterning of hair growth [4]. The literature describes the use of warm rosin and wax [6,7] or cold wax strips [8,9], although details on protocols and outcomes are often missing. In the attempts to reproduce those depilation methods, we faced wound development in all animals used. Skin wounds are described to alter hair growth [10,11] and, importantly, can affect animal welfare. Our new method for depilation using cold wax (*Veet minima*) followed by wax clearance with mineral oil, and the use of two protective sprays, *Douxo Calm Sérum* and *Cavilon*, led to a substantial decrease in wound frequency, and a notable reduction in wound severity. Therefore, this method has the potential to impact hair research, by excluding the confounding effects of wounds in hair cycle quantifications, in addition to improving animal welfare during experiments.

It is also relevant to mention the importance of using 7-week-old mice in the context of hair synchronization. The time-scale for the natural hair cycle in *C57BL/6J* mice is well established: 7-week-old mice hair is in telogen, progressing to anagen in the following weeks [2], although hair growth follows a wavy patterning across the body [4]. We found that 8-week-old mice already present small pigmented areas in the back skin, which we could not synchronize back to telogen by depilation. These pigmented patches introduce noise to the readout and should be avoided. Synchronization using 7-week-old mice guarantees that all hair follicles initiate anagen at the same time.

Whilst having wound issues upon depilation in the *C57BL/6J* mice, we tested other mice strains. *C57BL/6J* mice are particularly prone to skin conditions [12], and other dark hair strains, such as *Sv129* and the mixed background *B6129F1*, have not been characterized in terms of their hair cycle. After visual analysis of hair growth, we found that, at the same timepoint (days 7 and 9), the skin color of *Sv129* and *B6129F1* mice is darker than in *C57BL/6J* mice, and when hair starts to grow, *Sv129* and *B6129F1* hair is denser. Skin histomorphological analysis showed that *Sv129* and *B6129F1* skin is morphologically different from *C57BL/6J* skin: total skin thickness is higher in *Sv129*, and subcutaneous layer thickness is higher in both *Sv129* and *B6129F1*. Moreover, when in anagen, *Sv129* and *B6129F1* mice present increased number and size of hair follicles. However, concerning hair cycle kinetics, our data suggest that hair cycle proceeds at a similar pace in all analyzed strains. Together, these data indicate that hair density is higher in *Sv129* and *B6129F1* mice, albeit hair cycle kinetics is similar between all strains. Since *C57BL/6J* mice provide better contrast between lighter skin and darker hair, and the new protocol mitigates wound development, *C57BL/6J* mice are still the best model for hair research; nevertheless, *Sv129* and *B6129F1* mice hair cycle is now characterized, and can be used in specific circumstances.

## 5. Conclusions

This study discloses an optimized mouse depilation protocol that excludes the detrimental effects of skin injury in hair growth studies, and in addition, characterizes the hair cycle of distinct mouse strains fostering their use in hair growth pre-clinical studies.

## Figures and Tables

**Table 1:** Experimental animal information.

Figure	Mice Strain	Age	Number of Animals	Notes
Figure 1A,B	*C57BL/6J*	7 weeks-old	other methods n = 15	
			optimized method n = 10	Animals also used for Figure 1D
Figure 1D	*C57BL/6J*		n = 19	Animals also used for Figure 2 and Figure 3
	*B6129F1*		n = 17	
	*Sv129*		n = 17	
Figure 2 and Figure 3	*C57BL/6J*		n = 19	
	*B6129F1*		n = 17	
	*Sv129*		n = 17	

## Data Availability

The data supporting the findings are presented in the article. Any further queries regarding the data can be directed to J.M.
